# Sociodemographic and Health Determinants of Adipose Tissue Distribution in a Local Community from Eastern Poland: A Cross-Sectional Study

**DOI:** 10.3390/jcm14186642

**Published:** 2025-09-20

**Authors:** Maciej Polak, Grzegorz Józef Nowicki, Magdalena Kozela, Maciej Matyja, Barbara Ślusarska

**Affiliations:** 1 Department of Epidemiology and Population Studies, Medical College, Jagiellonian University, Skawińska 8 Str., 31-066 Krakow, Poland; maciej.1.polak@uj.edu.pl (M.P.); m.kozela@uj.edu.pl (M.K.); 2 Department of Family and Geriatric Nursing, Faculty of Health Sciences, Medical University of Lublin, Staszica 6 Str., 20-059 Lublin, Poland; barbara.slusarska@umlub.pl; 3 2nd Department of General Surgery, Medical College, Jagiellonian University, Jakubowskiego 2 Str., 30-688 Krakow, Poland; maciej.matyja@uj.edu.pl

**Keywords:** abdominal volume index (AVI), conicity index (C-index), weight-adjusted-waist index (WWI), public health

## Abstract

**Objectives:** The aim of this study was to assess the distribution of abdominal volume index (AVI) conicity index (C-Index) and weight adjusted waist index (WWI) attributes by sociodemographic and health characteristics in apparently healthy individuals (residents of the Janów Lubelski district in the eastern Poland). Additionally, the study examined whether sociodemographic and health characteristics differentiate the distribution of adipose tissue indicators in individuals with a normal body weight, defined as a BMI of less than 25 kg/m^2^. **Methods:** A total of 3752 apparently healthy respondents participated in the cross-sectional study. In order to determine the participants’ adipose tissue distribution, professionally trained nurses measured their anthropometric indices and interviewed them to assess the sociodemographic and health variables. **Results:** The study group’s mean values for anthropometric indices related to central adipose tissue distribution were as follows: C-Index 1.26 ± 0.088, AVI 18.28 ± 4.96 and WWI 10.63 ± 0.73. The three indices examined in multivariable analyses showed a significant relationship with age, gender, place of residence, education, living alone, smoking status, alcohol consumption and comorbidities (diabetes, hypertension and hypercholesterolemia). **Conclusions:** The study findings demonstrate a significant relationship between the level of specific anthropometric indices related to central adipose tissue distribution and sociodemographic and health-related variables. The employment of certain anthropometric indices related to central adipose tissue distribution, which are derived from waist circumference, can be beneficial in primary healthcare by potentially facilitating early prevention of cardiometabolic diseases.

## 1. Introduction

The relationship between socio-economic status (SES) and the prevalence of excess body weight is well documented, but varies by world region and gender [1]. Results of cross-sectional studies show that SES and obesity are closely associated with country economy. This relationship is more specific in women than in men [2]. The latest data from Europe confirms that socioeconomic disadvantages are associated with increased prevalence of overweight and obesity. Indeed, even within the European region, the variation in observed relationships by income is noticeable, as the results in countries of lower income are not fully consistent. In Bulgaria [3] and Hungary, the lowest SES was found to be connected with lower rates of overweight/obesity. The vast majority of longitudinal studies assessing the relationship between socioeconomic status and body weight, however, come from high-income countries [4]. In the systematic review by Newton et al. [1], a consistent relationship between lower life course SES and higher BMI, as well as obesity prevalence with waist circumference in women was found. Whereas, in men, only a positive association with waist circumference was confirmed.

The fact that differences in the relationship between SES and obesity depend on whether the BMI or waist circumference was taken into account indicates that not only excess body weight but also distribution of body fat should be considered. It was discovered that BMI may overstate or underestimate the socioeconomic gradient in obesity when compared to research that examine fat distribution or body composition [5,6]. Moreover, people with appropriate BMI may have high adipose tissue content, and using BMI alone to capture cardiometabolic risk may be insufficient as BMI may not be regarded as a biomarker of abdominal adiposity. The absence of adiposity distribution in obesity surveillance may not fully characterise the health risk associated with the worldwide obesity prevalence, especially when the prevalence of abdominal obesity is increasing [7].

In the response to the needs for more in-depth population studies on socioeconomic gradient in obesity, as well as the clinical need for a rapid assessment of the distribution of adipose tissue, a number of new scales based on anthropometric measurements have been developed. These allow the addressing of the problem of adipose tissue distribution without using additional advanced equipment [8]. Abdominal volume index (AVI), for example, was developed as a reliable and easy-to-calculate anthropometric instrument for estimation of overall abdominal volume [9]. Such an index has been shown to be strongly related to impaired glucose tolerance and diabetes mellitus. AVI is now recognised as one of the best indicators for assessing fat deposition in the abdominal region, having the strongest link with body fat percentage, waist circumference and waist to hip ratio. Because it is independent of weight, it is an excellent candidate to replace BMI [8]. Weight-adjusted-waist index (WWI) is defined as an adiposity index representing waist circumference. It has a poor correlation with BMI, and has a negative correlation with height to distinguish between the influence of height on the same waist [10]. It was created as a useful alternative marker of obesity and obesity-related severe health outcomes, and it is projected to provide simple information about persons at risk of cardiometabolic disease and associated mortality with a single measurement. Conicity index (C-Index) was based upon the idea that as people gain fat around their waists, the shape of their bodies appears to change from that of a cylinder to that of a “double cone” (two cones with a shared base) [11]. Conicity has been criticized for failing to consider the women’s obesity model. This is partly due to its mathematical model, which ranges from cylinder to double cone, and it has little sensitivity to women’s pear-shaped model of obesity, instead viewing it as a cylinder form [8]. However, it cannot be excluded that it may be an accurate predictor of body fat in men.

Considering income related differences in the association between SES and excess body weight as well as east-west health gap in Europe, the assessment of AVI, WWI and C-Index may provide a more nuanced understanding of socioeconomic gradient in obesity and its health implications. Enhanced understanding of these relationships in normal BMI populations might be interesting, as in lower income countries, even individuals with a normal BMI can reveal hidden cardiometabolic risks associated with central adiposity. As Central European populations exhibit diverse SES structures, more in-depth analysis of the role of fat distribution may contribute to more precise public health initiatives. The aim of the analysis was to assess the distribution of abdominal volume index (AVI) conicity index (C-Index) and weight adjusted waist index (WWI) by sociodemographic and health characteristics in apparently healthy individuals (residents of the Janów Lubelski district in the Eastern Poland). Additionally, in the course of the study, we determined whether sociodemographic and health characteristics differentiate the distribution of adipose tissue indicators in people with a normal body weight, i.e., with BMI below 25 kg/m^2^.

## 2. Materials and Methods

### 2.1. Study Design and Participants

Between 14 June 2015 and 20 March 2016, a prevention and health promotion programme named “Take your health to heart” (“Weź sobie zdrowie do serca”) was carried out in the Janów District, Lubelskie Province, eastern Poland, during which data was collected and utilised for scientific research. The programme, which was funded by the Norwegian Financial Mechanism’s “PL13 Social Inequalities in Health 2009–2014 Programme”, was implemented in the Janów District as the funding covered local communities with high standardised mortality ratios (SMRs), and this region had a high prevalence of cardiovascular disease [12]. The programme’s details and the beneficiaries’ group are described in detail elsewhere [13]. In summary, a total of 4 040 individuals applied to participate in the study, with a 21.45% participation rate among all eligible participants. The inclusion criteria were as follows: (1) age between 35 and 64 years; (2) no history of cardiovascular incident; (3) no diagnosis of coronary artery disease; (4) informed consent to participate in the study. A total of 421 individuals (10.42%) were not included in the study as they did not meet the inclusion criteria. A history of cardiovascular incident (myocardial infarction or stroke) or a diagnosis of coronary artery disease disqualified 288 participants from taking part in further studies. The cohort consisted of 3752 participants that fulfilled the inclusion criteria.

### 2.2. Data Collection and Ethics Approval

A team of professionally trained nurses collected data and took anthropometric measurements based on strictly defined study protocol. Each study participant filled out a survey and had their anthropometric measurements taken (weight, height, waist and hip circumferences). The ethical approval was issued by the Bioethics Committee at the Medical University of Lublin (decision number: KE-0254/112/2014) and the study was carried out in accordance with the Declaration of Helsinki. Each participant provided his or her written informed consent to take part in the study.

### 2.3. Anthropometric Measurements and Determination of Anthropometric Indices Related to Overweight and Obesity

All participants’ height, weight, waist circumference and hip circumference were measured. The subject’ body weight (without shoes or outer clothes) was measured to the nearest 0.1 kg on a platform scale, and their height was measured to the nearest 0.1 cm using an altimeter. In the subsequent stage, BMI, defined as body weight in kilograms (kg) divided by height in meters squared (kg/m^2^), was calculated for each study subject. According to the WHO guidelines, the following BMI classification system was adopted: underweight (BMI ≤ 18.5 kg/m^2^), normal (BMI 18.5–24.9 kg/m^2^), overweight (25.0–29.9 kg/m^2^) and obese (BMI ≥ 30 kg/m^2^) [14]. A rigid measuring tape was used to measure waist circumference (WC). This was classified as the area between the lowest ribs and the top border of the iliac crest and hip circumference (HC)—at the level of the greater trochanter of the femur. The participant exhaled air and spread the legs apart by 25 to 30 cm to distribute body weight when measurements were taken. The measurements were collected when the study subject was standing [15].

Based upon anthropometric measurements, Conicity index (C-Index), abdominal volume index (AVI) and weight-adjusted-waist index (WWI) were calculated according to the formulas shown below [16]. Higher values are considered unfavourable.

$$C-Index=\frac{WC\;\left(m\right)}{0.109\sqrt{\frac{body\;weight\;\left(kg\right)}{height\;\left(m\right)}}}$$

$$AVI=\frac{{2x\left(WC\;\left(cm\right)\right)}^{2}+{0.7\times \left(WC\;\left(cm\right)-HC\;\left(cm\right)\right)}^{2}}{1000}$$

$$WWI=\frac{WC\;\left(cm\right)}{\sqrt{weight\;\left(kg\right)}}$$

### 2.4. Covariates

A standard survey questionnaire was utilised in order to collect information, such as age, gender, place of residence, education, marital status, living alone, smoking status, frequency of alcohol consumption and diagnosed chronic diseases.

The following categories of smoking status were established: never-smoker (if the respondent had never smoked any sort of tobacco products), former smoker (if the respondent had stopped smoking some sort of tobacco product at least one month prior to the study) and active smoker (the respondent smoked at least one cigarette a day or had smoked some sort of tobacco product in the preceding month).

Additionally, the study participants were asked about their alcohol intake in the year preceding the study. Respondents were asked how frequently they consumed 1–2 standard doses of alcohol, each containing 10 g of pure ethyl alcohol. The following options were available: “I do not drink alcohol at all”, “I drink alcohol less than once a month”, “I drink alcohol once a month to once a week” and “I drink alcohol more than once a week”.

To assess the physical activity, the respondents were requested to indicate whether they regularly engage in uninterrupted physical activity at least five times a week for 30 min. Adequate physical activity was defined as ≥150 min of uninterrupted activity per week.

The occurrence of depressive symptoms (DS) in the past 2 weeks was assessed through the Patient Health Questionnaire-9 (PHQ-9) [17]. The overall scale scores are calculated as the sum of 9 items (range between 0 and 27). A PHQ-9 score of 10 or higher indicates a risk of depressive symptoms. The PHQ-9 questionnaire had the optimal internal consistency in the study group, which is shown in another research paper [18].

### 2.5. Statistical Analysis

Numerical data is expressed as mean (M) with standard deviation (SD). The Shapiro-Wilk test was utilised to assess conformity with a normal distribution. The means of C-Index, AVI, WWI were compared between groups using t test or analysis of variance (ANOVA). The Pearson correlation was used to assess the relationship between numerical variables. Multivariable linear regression with backward elimination method (*p* < 0.1) was used to find significant predictors of the C-Index, AVI and WW. The results showed 95% confidence interval (CI) for a beta coefficient. The coefficient of determination (R2) was used to describe a goodness-of-fit for performed linear regression models. Two-sided *p*-values < 0.05 were recognised statistically significant. Analyses were conducted through IBM Corp. software released in 2022 (IBM SPSS Statistics for Windows; version 28.0.; IBM Corp, Armonk, NY, USA).

## 3. Results

### 3.1. General Characteristics of the Study Participants

Table 1 shows the characteristics of the study group (*n* = 3752). The mean age in the study group was 51.92 ± 8.15 years. More than half of the 3752 respondents (58.66%, *n* = 2201) were female, residing in rural areas (66.87%, *n* = 2509) and being in relationships (87.95%, *n* = 3300). Only 15.8% admitted smoking some sort of tobacco product and 4.5% of all respondents stated consuming 10–20 g of pure alcohol more than once per week. In terms of disease prevalence, 3.92% had diabetes, 25.43% had hypertension and 9.06% had hypercholesterolemia. According to anthropometric indices related to overweight and obesity, the study group’s mean BMI was 28.55 ± 4.49, whereas 24.22% of all respondents had normal BMI. In turn, the mean values of anthropometric indices related to adipose tissue distribution were as follows: C-Index 1.26 ± 0.088, AVI 18.28 ± 4.96 and WWI 10.63 ± 0.73.

### 3.2. Relationship Between Sociodemographic and Health Variables and Anthropometric Indices Related to Adipose Tissue Distribution

Table 2 shows the relationship between sociodemographic and health variables and the C-Index, AVI and WWI. All of the anthropometric indices related to adipose tissue distribution were positively correlated with age. As compared to respondents with secondary and higher education, respondents with primary and vocational education, rural residents and men had significantly higher mean values of the C-Index, AVI and WWI. Similarly, living alone, being a former smoker or active smoker, consuming 10–20 g of alcohol more than once a week and between once a month to once a week and having diabetes, hypertension or hypercholesterolaemia were associated with higher values of the three indices studied. In addition, the assessment of the occurrence of depressive symptoms through the PHQ-9 questionnaire revealed that respondents with a score of 10 or higher had significantly higher values of AVI and WWI, while married or single respondents had significantly lower values of WWI than did widows and widowers.

Table 3 presents the relationship between selected sociodemographic and health variables and the C-Index, AVI and WWI values in the subgroup of patients with normal BMI that ranged between 18.5 and 24.99 kg/m^2^ (*n* = 905). As in the entire study group, age, gender, education, smoking status, alcohol consumption and the presence of hypertension were all significantly correlated with anthropometric indices related to adipose tissue distribution. However, we found that in the subgroup of subjects with normal BMI, those who engaged in 150 min of weekly physical activity had noticeably higher values of C-Index, AVI and WWI. In contrast, there was no relationship between the place of residence, marital status, living alone and having diabetes and hyperlipidaemia. In addition, among the subgroup of subjects with normal BMI, those who scored less than 10 on the PHQ-9 questionnaire had a significantly higher C-Index.

### 3.3. Multivariable Association Between Anthropometric Indices Related to Adipose Tissue Distribution and Socioeconomic and Health Factors

Table 4 presents the significant predictors of C-Index, AVI and WWI obtained in linear regression. Age, gender, education, prevalence of diabetes and hypertension were significantly associated with all indices related to adipose tissue distribution. A positive correlation was found between age and C-Index, AVI and WWI values. In addition, men had higher mean values of C-Index, AVI and WWI compared to women. Moreover, individuals with diabetes and hypertension had higher mean values than those without these medical conditions. As compared to respondents with only a primary education, those with vocational, secondary and higher education had lower values for anthropometric indices related to adipose tissue distribution.

Additionally, rural respondents as compared to urban respondents, individuals who declared consuming 10–20 g of alcohol once a month to once a week as compared to non-drinkers, and respondents with diagnosed hypercholesterolaemia as compared to respondents without this medical condition had significantly higher, AVI and WWI values. It should also be noted that active smokers had a significantly lower AVI value as compared to never-smokers, while a higher AVI value was found among former smokers as compared to never-smokers. Finally, respondents with PHQ-9 ≥ 10 had a higher AVI value as compared to respondents with a PHQ-9 questionnaire less than 10.

Table 5 shows the significant predictors of C-Index, AVI and WWI obtained in linear regression in a subgroup of respondents with normal BMI. As in the entire study group, the C-Index, AVI and WWI values were positively correlated with age. Moreover, the values of these indices were significantly higher in individuals with diagnosed hypertension. Furthermore, our research showed that respondents with only a primary education had higher WWI and C-Index values as compared to those with vocational, secondary and higher education. In turn, persons living alone had a higher AVI value. Finally, we found that never-smokers had significantly lower C-Index and AVI as compared to active smokers or former smokers. Additionally, when assessing the occurrence of depressive symptoms through the PHQ-9 questionnaire, respondents with a score of 10 or higher had lower C-Index and higher AVI than those with a score below 10.

## 4. Discussion

This study assessed the distribution of AVI, C-Index and WWI values related to adipose tissue distribution in relation to sociodemographic and health determinants in an apparently healthy local population. In addition, we determined whether sociodemographic and health variables differentiate the distribution of the aforementioned indices in individuals with normal BMI. The importance of the regional distribution of adipose tissue, especially central adiposity, which cannot be measured by BMI, has drawn the attention of researchers in recent years as it has become evident that different regional locations of adipose tissue have diverse metabolic implications and are more significant than total body fat mass [19]. Our study results showed that, for both the entire study population and the subgroup of respondents with normal BMI, the values of the C-Index, AVI and WWI were significantly correlated with the related sociodemographic and health variables.

A new syndrome, known as normal-weight obesity (NWO), occurs in individuals with a normal BMI < 25 kg/m^2^ but with excessive body fat [20], and research has shown that low physical activity, being a former smoker and alcohol consumption are related to NWO, but not with slimness [21]. It should be noted that NWO may occur at any age and is associated with a higher risk of poor skeletal strength, metabolic disorders and the development of the metabolic syndrome [22,23].

Lifestyle changes and improved socioeconomic situations may explain why anthropometric indices related to overweight and obesity are increasing in the Western world [24]. There have been many studies examining the impact of sociodemographic variables. These have the intent of bringing to light the most effective lifestyle habits for maintaining a healthy body weight throughout the typical human life span. There is thus a wealth of research indicating that SES is the most important variable in maintaining a healthy body weight in the entire course of life.

Poland, as a high-income country in Central and Eastern Europe, has changed significantly over the last three decades [25]. Since joining the European Union (EU) in 2004, numerous social and economic changes have impacted dietary habits [26]. In accordance with the Central Statistical Office, Poland’s GDP was 70% higher in 2020 than in 2005, that is the year following Poland’s EU accession [27].

The study’s first finding was that there is a correlation between sociodemographic variables and particular anthropometric indices related to central adiposity. We established that AVI, C-Index and WWI were positively related to age, which is consistent with previous research [28,29]. Age is a determinant of lifestyle. Recent studies have also shown that middle-aged and older people consume more alcohol than is deemed safe and healthy [30]. However, a large number of people in this age group have given up smoking. Although our study revealed that the male sex is linked to higher values of the anthropometric indices, lifestyle habits have changed throughout the past few decades and are more equal between the sexes than in previous generations, which has implications for body weight and composition [31].

Overweight and obesity are key risk factors for many noncommunicable diseases. Our study showed that the anthropometric parameters under consideration were higher in individuals with comorbidities. In addition, the burden of disease increases with age. Older people who are overweight or obese may have more severe health problems that affect their healthcare use, disability and fitness to work [32].

Furthermore, we found that education has a significant relationship with the anthropometric indices under consideration. The above may result from higher socioeconomic status and income level among individuals with higher education, which may translate into more healthy dietary habits and increased levels of physical activity [33]. As has been noted in earlier research, this correlation should be interpreted cautiously as it might be connected with selection bias [34]. Given that older respondents with higher education and greater income levels as compared to non-participants are generally more likely to participate in epidemiological studies, selection bias may partially affect some of the relationships found.

According to our research, AVI is positively correlated with the occurrence of depressive symptoms. Depression and obesity are linked by a number of common biological mechanisms, ranging from genetics, to changes in various immune-inflammatory and metabolic systems [35]. In accordance with the Diagnostic and Statistical Manual of Mental Disorders, 4th edition (DSM-IV), increased appetite and weight gain are symptoms of depression, which may explain the results we obtained, as well as the findings of other studies [36].

It is necessary to discuss two results obtained in one-dimensional analyses in the subgroup of individuals with normal BMI values. We discovered that individuals who reported engaging in physical activity more frequently had considerably higher C-Index, AVI and WWI scores. In turn, in the same subgroup, those who did not show any signs of depression had significantly higher C-Index scores. According to a recent meta-analysis by Armstrong et al. [37], the relationship between the duration and intensity of physical exercises and the WC index, which was utilised in our study to calculate the analysed indices associated with central fat distribution, is unclear. It should be noted that the authors of the meta-analysis themselves suggest that this relationship should be further studied. The second potential mechanism that could explain our results may be associated with the impact of confounding variables in univariable analyses, as in multivariable analyses, after taking confounding variables into consideration, physical activity was not a significant predictor in the subgroup of individuals with a normal BMI. In turn, respondents with a score of 10 or higher on the PHQ-9 questionnaire had a lower C-Index and a higher AVI. Therefore, further research in this area should be considered.

We then decided to examine whether sociodemographic variables differentiate the distribution of specific anthropometric indices related to central adiposity in the subgroup of subjects with normal BMI. Our study results showed that, in the subgroup of individuals with normal BMI, persons living alone had significantly higher values of these indices. However, in terms of sociodemographic characteristics, place of residence was not significantly related to the anthropometric variables examined. In terms of health variables, the declared alcohol consumption did not differentiate the values of the anthropometric indices analysed in the subgroup of subjects with normal BMI. With regard to co-morbidities, only subjects with hypertension had higher C-Index, AVI and WWI values.

A few explanatory mechanisms can, therefore, be indicated. Along with Poland’s economic growth and urbanisation of lifestyles, the prevalence of overweight and obesity among rural and urban residents is rising, while the disparity between these two settlements is narrowing significantly. In the past, living in rural areas required more physical activities in daily life, for example, in farming practices. However, due to the now widespread use of agricultural machinery, the transition from small to large farms and the fact that an increasing number of rural residents work in white-collar jobs, the disparity regarding the risk of central adiposity is being balanced.

We also found that higher values of certain anthropometric indices related to central adiposity were associated with being single rather than being in a relationship. Previous research has indicated that married men were less likely to smoke and consume more vegetables and cereal fibre in their diets than divorced men. Moreover, as compared to married men, divorced men smoked more frequently, consumed more alcohol, yet were engaged in more physical activity [38]. It can be concluded that marital status has a significant impact on lifestyle choices that affect the prevalence of overweight and obesity, though this impact may vary by culture.

Although hypertension is common in obese people, its pathogenesis has been only partially explained. A number of factors, including leptin, the sympathetic nervous system, the renin-angiotensin-aldosterone system, the neuroendocrine nervous system and renal compression [39,40,41,42], have been suggested as potential causes. In view of the fact that obesity is a risk factor for numerous illnesses [43], some patients may initially develop obesity before other non-communicable diseases, such as hypertension. However, the aforementioned issues require further research due to the study’s cross-sectional design. Similar to our study, Owolabi et al. [44] found that in the subgroup of subjects with normal BMI, central adiposity was associated with hypertension, but not with diabetes. Our study and the research mentioned above stress how important it is to incorporate central adiposity measurements into clinical patient assessment in order to reduce cardio-metabolic risk factors. In order to lower the rising prevalence of chronic diseases, which have a significant impact on an already overburdened healthcare system, reducing central adiposity should be a top priority.

This is the first study that we are aware of that assesses the relationship between sociodemographic variables and particular anthropometric indices related to central adiposity in a population from Central and Eastern Europe. In undertaking it, correlations were examined in a well-defined ethnically homogeneous group from the general population, as well as in a subgroup of individuals with normal BMI. Additionally, we employed standardised test methods based on detailed protocols to reduce the risk of a systematic error. In order to determine the prevalence of depressive symptoms in our study population, we utilised the PHQ-9 questionnaire, which demonstrated good reliability [18].

In spite of these advantages, this study has certain limitations. Firstly, since this is a cross-sectional study, it is far from certain that the observed differences in anthropometric indices are due to variations in sociodemographic characteristics. Still, it appears unlikely that these relationships operate in the opposite direction. Secondly, the metrics are based on anthropometric measurements and specific assumptions rather than an objective examination of adipose tissue distribution. However, the literature has shown that they are highly correlated with the results of studies examining the actual adipose tissue distribution. The determination of a relationship between these metrics and key socioeconomic and health characteristics may help spread a quick and easy-to-use tool for assessing adipose tissue distribution. Thirdly, as compared to the general population, the values of certain anthropometric indices related to central adiposity may also be overestimated since the respondents had a higher cardiovascular risk, most likely as a result of risk factors, such as poor diet and physical inactivity. This should, however, have no significant impact on the observed relationships with sociodemographic characteristics. Fourthly, although we have included significant characteristics in the model that could explain the variation in the anthropometric indices, it is unclear whether this variation is caused by residual confounding from another unmeasured factor. Fifthly, despite an in-depth review of the literature, the authors could not find any validation studies of the C-Index, AVI and WWI in the European population. However, validation studies in adult and paediatric populations in Brazil [45,46] and young adults in China [47] have demonstrated their reliability in assessing central obesity. Finally, sixthly, we did not utilise dual-energy X-ray absorptiometry (DXA) to assess body fat in our study. However, in a population study of this size, using DXA could be challenging.

## 5. Conclusions

The study findings show a considerable relationship between the level of specific anthropometric indices related to central adipose tissue distribution and sociodemographic and health-related variables. The level of specific anthropometric indices related to central adiposity increases with age and is higher in men, rural residents, single people and individuals with poorer education. Moreover, among the health variables examined in this study, we found that certain anthropometric indices related to central adiposity are higher in active or former smokers, those who declare consuming more alcohol, have chronic illnesses and experience depressive symptoms. The employment of certain anthropometric indices related to central adipose tissue distribution, which are derived from waist circumference (WC), can be useful in primary healthcare for NWO assessment and may facilitate early prevention of cardiometabolic diseases.

## Figures and Tables

**Table 1:** Characteristics of the study group.

Variables	Total (*n* = 3752) ^a^
Demographic data:	
Age [years]	51.92 ± 8.15
Sex	
Female	2201 (58.6)
Male	1551 (41.35)
Place of residence	
Rural	2509 (66.86)
Urban	1243 (33.14)
Education	
Primary	413 (11.01)
Vocational	1390 (37.06)
Secondary	1204 (32.07)
Higher	745 (19.86)
Marital status	
Married	3300 (87.95)
Single (bachelor/bachelorette)	272 (7.25)
Widow/widower	180 (4.8)
Living alone (Yes)	174 (4.64)
Health data:	
Smoking status:	
Active smoker	595 (15.86)
Former smoker	782 (20.84)
Never-smoker	2375 (63.3)
Alcohol consumption:	
No or less than once a month	3345 (89.2)
Between once a month and once a week	237 (6.3)
More than once a week	170 (4.5)
Physical activity (performing ≥ 150 min of activity each week)	1604 (42.76)
Co-morbidities:	1088 (29)
Diabetes	147 (3.92)
Hypertension	954 (25.43)
Hypercholesterolemia	340 (9.06)
PHQ-9 (≥10)	605 (16.1)
BMI [kg/m^2^]:	
Normal [18.5–24.99 kg/m^2^]	905 (24.22)
Overweight [25–29.99 kg/m^2^]	1510 (40.41)
Obesity [≥30 kg/m^2^]	1322 (35.37)
BMI	28.55 ± 4.98
C-Index	1.26 ± 0.088
AVI	18.28 ± 4.96
WWI	10.63 ± 0.73

Notes: ^a^ Mean ± SD or percentages are listed. Abbreviations: PHQ-9: Patient Health Questionnaire; BMI: body mass index; C-Index: conicity index; AVI: abdominal volume index; WWI: weight-adjusted-waist index.

**Table 2:** Association between selected sociodemographic and health variables and anthropometric indices related to adipose tissue distribution.

Variables	C-Index	*p*	AVI	*p*	WWI	*p*
Demographic data:						
Age [years]	r = 0.33	<0.001	r = 0.27	<0.001	r = 0.4	<0.001
Sex:						
Female	1.23 ± 0.09	<0.001	16.87 ± 4.77	<0.001	10.56 ± 0.81	<0.001
Male	1.3 ± 0.07		20.28 ± 4.54		10.73 ± 0.59	
Place of residence:						
Rural	1.26 ± 0.09	<0.001	18.96 ± 4.96	<0.001	10.66 ± 0.73	<0.001
Urban	1.25 ± 0.09		17.74 ± 4.93		10.56 ± 0.73	
Education:						
Primary	1.3 ± 0.08	<0.001	20.33 ± 5.13	<0.001	11.07 ± 0.66	<0.001
Vocational	1.27 ± 0.08		18.85 ± 4.86		10.72 ± 0.66	
Secondary	1.25 ± 0.08		18.02 ± 4.78		10.58 ± 0.71	
Higher	1.22 ± 0.09		16.51 ± 4.73		10.3 ± 0.76	
Marital status:						
Married	1.26 ± 0.09	0.98	18.31 ± 4.91	0.46	10.62 ± 0.73	<0.001
Single (bachelor/bachelorette)	1.26 ± 0.09		18.08 ± 5.77		10.61 ± 0.76	
Widow/widower	1.26 ± 0.08		18.06 ± 4.7		10.87 ± 0.75	
Living alone						
No	1.26 ± 0.09	0.004	18.24 ± 4.91	0.01	10.62 ± 0.73	<0.001
Yes	1.27 ± 0.09		19.22 ± 5.83		10.86 ± 0.77	
Health data:						
Smoking status:						
Active smoker	1.27 ± 0.08	<0.001	18.02 ± 4.86	<0.001	10.64 ± 0.67	<0.001
Former smoker	1.27 ± 0.08		19.39 ± 4.94		10.71 ± 0.68	
Never-smoker	1.24 ± 0.09		17.98 ± 4.92		10.6 ± 0.75	
Alcohol consumption:						
No or less than once a month	1.25 ± 0.09	<0.001	18.07 ± 4.9	<0.001	10.62 ± 0.74	0.02
Between once a month and once a week	1.29 ± 0.07		20.25 ± 5.43		10.74 ± 0.64	
More than once a week	1.29 ± 0.07		19.62 ± 4.78		10.7 ± 0.58	
Physical activity (performing ≥ 150 min of activity each week):						
No	1.26 ± 0.09	0.72	18.39 ± 5.13	0.113	10.63 ± 0.75	0.89
Yes	1.26 ± 0.08		18.14 ± 4.74		10.63 ± 0.7	
Co-morbidities:						
Diabetes:						
No	1.25 ± 0.09	<0.001	18.08 ± 4.8	<0.001	10.61 ± 0.73	<0.001
Yes	1.32 ± 0.08		23.19 ± 6.3		11.17 ± 0.69	
Hypertension:						
No	1.24 ± 0.09	<0.001	17.44 ± 4.66	<0.001	10.51 ± 0.72	<0.001
Yes	1.29 ± 0.08		20.75 ± 5.0		10.97 ± 0.66	
Hypercholesterolemia:						
No	1.25 ± 0.09	<0.001	18.04 ± 4.9	<0.001	10.59 ± 0.73	<0.001
Yes	1.29 ± 0.08		20.68 ± 5.0		11 ± 0.67	
PHQ-9 ≥ 10						
No	1.26 ± 0.08	0.17	18.2 ± 4.88	0.02	10.61 ± 0.71	<0.001
Yes	1.26 ± 0.1		18.71 ± 5.38		10.74 ± 0.84	

Abbreviations: As in Table 1; r: correlation coefficient.

**Table 3:** Association between selected sociodemographic and health variables and anthropometric indices related to adipose tissue distribution in a subgroup of respondents with normal BMI (*n* = 905).

Variables	C-Index	*p*	AVI	*p*	WWI	*p*
Demographic data:						
Age [years]	r = 0.26	<0.001	r = 0.21	<0.001	r = 0.3	<0.001
Sex:						
Female	1.17 ± 0.08	<0.001	12.31 ± 1.95	<0.001	10 ± 0.72	<0.001
Male	1.26 ± 0.06		15.15 ± 2.01		10.43 ± 0.57	
Place of residence:						
Rural	1.2 ± 0.09	0.066	13.28 ± 2.3	0.06	10.16 ± 0.71	0.13
Urban	1.19 ± 0.08		12.98 ± 2.43		10.09 ± 0.69	
Education:						
Primary	1.25 ± 0.08	<0.001	14.35 ± 2.36	<0.001	10.61 ± 0.64	<0.001
Vocational	1.21 ± 0.08		13.67 ± 2.32		10.24 ± 0.6	
Secondary	1.19 ± 0.08		12.99 ± 2.29		10.12 ± 0.69	
Higher	1.17 ± 0.09		12.5 ± 2.24		9.91 ± 0.75	
Marital status:						
Married	1.19 ± 0.09	0.92	13.19 ± 2.36	0.65	10.13 ± 0.71	0.23
Single (bachelor/bachelorette)	1.19 ±0.08		13.06 ± 2.4		10.08 ± 0.67	
Widow/widower	1.2 ± 0.08		12.86 ± 2.18		10.32 ± 0.69	
Living alone						
No	1.2 ± 0.09	0.52	13.19 ± 2.37	0.072	10.13 ± 0.71	0.95
Yes	1.2 ± 0.06		12.46 ± 1.89		10.14 ± 0.57	
Health data:						
Smoking status:						
Active smoker	1.23 ± 0.08	<0.001	13.94 ± 2.34	<0.001	10.32 ± 0.6	<0.001
Former smoker	1.21 ± 0.09		13.66 ± 2.62		10.19 ± 0.71	
Never-smoker	1.18 ± 0.09		12.76 ± 2.2		10.05 ± 0.72	
Alcohol consumption:						
No or less than once a month	1.19 ± 0.09	<0.001	13.04 ± 2.32	<0.001	10.1 ± 0.71	<0.001
Between once a month and once a week	1.24 ± 0.07		14.42 ± 2.19		10.41 ± 0.58	
More than once a week	1.24 ± 0.07		14.18 ± 2.63		10.39 ± 0.59	
Physical activity (performing ≥ 150 min of activity each week):						
No	1.19 ± 0.09	0.013	13.03 ± 2.39	0.05	10.07± 0.73	0.008
Yes	1.2 ± 0.08		13.33 ± 2.31		10.2 ±0.67	
Co-morbidities:						
Diabetes:						
No	1.19 ± 0.09	0.42	13.16 ± 2.36	0.3	10.13 ± 0.7	0.32
Yes	1.22 ± 0.08		13.93 ± 1.61		10.35 ±0.72	
Hypertension:						
No	1.19 ± 0.08	<0.001	13.07 ± 2.33	<0.001	10.1 ± 0.69	<0.001
Yes	1.24 ± 0.09		14.16 ± 2.41		10.5 ± 0.76	
Hypercholesterolemia:						
No	1.19 ± 0.87	0.81	13.16 ± 2.36	0.91	10.13 ± 0.71	0.31
Yes	1.2 ± 0.78		13.22 ±2.09		10.29 ± 0.64	
PHQ-9 ≥ 10						
No	1.2 ± 0.08	0.03	13.22 ± 2.33	0.078	10.15 ± 0.66	0.12
Yes	1.18 ± 0.11		12.83 ± 2.51		10.04 ± 0.94	

Abbreviations: As in Table 2.

**Table 4:** Significant predictors of the C-Index, AVI and WWI levels.

Variables	C-Index			R^2^	AVI			R^2^	WWI			**R^2^**
	b	SE	*p*		b	SE	*p*		b	SE	***p***	
Demographic data:				30%				27%				23%
Age [years]	0.02	0.0002	<0.001		0.089	0.01	<0.001		0.026	0.001	<0.001	
Sex (reference category: Female):												
Male	0.06	0.003	<0.001		3.19	0.155	<0.001		0.099	0.023	<0.001	
Place of residence (reference category: Urban):												
Rural	0.004	0.003	0.098		0.602	0.16	<0.001		0.05	0.024	0.038	
Education (reference category: Primary):												
Vocational	−0.018	0.004	<0.001		−0.781	0.244	0.001		−0.186	0.037	<0.001	
Secondary	−0.03	0.004	<0.001		−1.027	0.254	<0.001		−0.291	0.038	<0.001	
Higher	−0.042	0.005	<0.001		−1.593	0.29	<0.001		−0.426	0.044	<0.001	
Health data:												
Smoking status (reference category: Never-smoker):												
Active smoker	0.003	0.003	0.025		−0.766	0.201	<0.001		-	-	-	
Former smoker	0.007	0.003	0.314		0.423	0.18	0.018		-	-	-	
Alcohol consumption (reference category: No or less than once a month):												
Between once a month and once a week	0.01	0.005	0.044		0.839	0.295	0.004		0.091	0.045	0.041	
More than once a week	0.011	0.006	0.059		0.347	0.347	0.472		0.076	0.052	0.144	
Co-morbidities:												
Diabetes (reference category: No):												
Yes	0.026	0.006	<0.001		2.603	0.374	<0.001		0.201	0.056	<0.001	
Hypertension (reference category: No):												
Yes	0.028	0.003	<0.001		2.354	0.178	<0.001		0.232	0.027	<0.001	
Hypercholesterolemia (reference category: No):												
Yes	-	-	-		0.542	0.259	0.036		0.089	0.039	0.022	
PHQ-9 ≥ 10 (reference category: No):												
Yes	-	-	-		0.446	0.192	0.02		-	-	-	

Abbreviations: PHQ-9: Patient Health Questionnaire; b: regression coefficients; SE: standard error; R^2^: coefficient of determination.

**Table 5:** Significant predictors of the C-Index, AVI and WWI levels in a subgroup of respondents with normal BMI (*n* = 905).

Variables	C-Index			R^2^	AVI			R^2^	WWI			**R^2^**
	b	SE	*p*		b	SE	*p*		b	SE	***p***	
Demographic data:				30%				34%				17%
Age [years]	0.001	0.0003	<0.001		0.031	0.008	<0.001		0.017	0.003	<0.001	
Sex (reference category: Female):												
Male	0.077	0.006	<0.001		2.685	0.148	<0.001		0.305	0.05	<0.001	
Education (reference category: Primary):												
Vocational	−0.018	0.01	0.06		-	-	-		−0.217	0.09	0.017	
Secondary	−0.023	0.011	0.03		-	-	-		−0.237	0.09	0.011	
Higher	−0.033	0.011	0.002		-	-	-		−0.368	0.1	<0.001	
Living alone (reference category: No):												
Yes	-	-	-		−0.956	0.335	0.004		-	-	-	
Health data:												
Smoking status (reference category: Never-smoker):												
Active smoker	0.014	0.007	0.048		0.188	2.85	0.004		-	-	-	
Former smoker	0.013	0.006	0.045		0.164	0.734	0.463		-	-	-	
Co-morbidities:												
Hypertension (reference category: No):												
Yes	0.0347	0.009	<0.001		0.234	3.596	<0.001		0.281	0.078	<0.001	
PHQ-9 ≥ 10 (reference category: No):												
Yes	−0.018	0.007	0.009		0.184	−1.667	0.096		-	-	-	

Abbreviations: As in Table 4.

## Data Availability

The datasets used and/or analysed during the current study are available from the corresponding author on reasonable request.
